# Pressurized IntraPeritoneal Aerosol Chemotherapy vs. intravenous chemotherapy for unresectable peritoneal metastases secondary to platinum resistant ovarian cancer – study protocol for a randomized control trial

**DOI:** 10.1515/pp-2018-0111

**Published:** 2019-03-02

**Authors:** S. P. Somashekhar, K. R. Ashwin, Amit Rauthan, Kumar C. Rohit

**Affiliations:** Consultant Surgical & Gynec. Onco & Robotic Surgeon, HIPEC Super Specialist, Manipal Comprehensive Cancer Center, Manipal Hospital, Bangalore, India; Bangalore, India

**Keywords:** ovarian cancer, palliative chemotherapy, peritoneal metastasis, platinum resistant ovarian cancer, Pressurized Intraperitoneal Aerosol Chemotherapy, response evaluation criteria in solid tumours

## Abstract

**Background:**

Despite optimal surgery and appropriate first-line chemotherapy, ∼70–80 % of patients with epithelial ovarian cancer will develop disease relapse. The prognosis is poor especially for women with Platinum resistant ovarian cancer. The standard treatment for these groups of patients is non-platinum-containing chemotherapy like taxanes, anthracyclines, gemcitabine, topotecan, and trabectedin. These drugs in various combinations and sequences provide modest survival or symptomatic benefit but with significant side effects. Pressurized IntraPeritoneal Aerosol Chemotherapy (PIPAC) is a minimally-invasive drug-delivery technique specifically addressing limited tissue penetration and poor drug distribution with promising results. PIPAC is a novel method of delivering normothermic chemotherapy into the abdominal cavity as an aerosol under pressure. This concept seems to enhance the effectiveness of intra peritoneal chemotherapy by taking advantage of the physical properties of gas and pressure by generating an artificial pressure gradient and enhancing tissue uptake and distributing drugs homogeneously within the closed and expanded peritoneal cavity. Thus, due to the high local bioavailability during PIPAC, the chemotherapy dosage can be reduced which in turn largely prevents systemic side effects and organ toxicity.

**Methods:**

The study aims to investigate the therapeutic efficacy measured as objective tumour response according to Response Evaluation Criteria in Solid Tumours (RECIST) criteria, of PIPAC in comparison with conventional Intravenous chemotherapy for women with recurrent platinum resistant ovarian cancer with peritoneal metastasis (PM). Consecutive patients diagnosed with PM secondary to platinum-resistant ovarian cancer will be randomized to PIPAC group or IV chemotherapy group. The primary objective of this study is to determine the efficacy after three cycles of PIPAC with cisplatin and doxorubicin in comparison with six cycles of systemic chemotherapy. The secondary outcome measures include morbidity and mortality, overall survival and disease specific survival. Analysis is by intention to treat.

**Aim:**

Assess the objective tumour response of PIPAC in comparison with systemic intravenous chemotherapy for women with platinum-resistant ovarian cancer.

**Study type:**

Prospective randomized control intervention trial.

**Intervention model:**

IV Chemotherapy group (Control group) PIPAC group (Experimental group)

**Masking:**

Open label.

**Primary purpose:**

Treatment.

**Sample size:**

Calculated sample size is 97 and rounded to 100. For each treatment group sample size of 50 will be considered.

**Primary outcome criteria:**

Objective tumour response according to Response Evaluation Criteria in Solid Tumours (RECIST) criteria version 1.1.

**Secondary outcome criteria**

1. Morbidity;
2. Disease-specific survival (months between inclusion and death due to ovarian cancer);
3. OS (months between inclusion and death due to any cause);
4. CA 125 levels.

**Discussion:**

PIPAC in women with platinum resistant ovarian PM has good response owing to superior tissue penetration and better drug distribution. The procedure is safe and well tolerated owing it to its minimal invasiveness. Typical side-effects of systemic chemotherapy, such as alopecia, peripheral neurotoxicity, nausea and myelosuppression are absent. We expect reduction of ascites with symptomatic relief and CA 125 levels. PIPAC is a novel technique for selected patients with platinum resistant ovarian PM and further investigation in comparative clinical trials with conventional chemotherapy will establish its role as a good palliative treatment option.

**Ethics committee approval:**

Obtained.

**Status:**

Recruiting.

**Trial registration number:**

REF/2018/08/021223 Registered on Clinical Trials Registry – India (CTRI); www.ctri.nic.in

## Background

Epithelial ovarian cancer is an aggressive malignancy and it is most frequently diagnosed in an advanced disease stage [1]. Currently, standard primary therapy for advanced disease involves the combination of surgery and systemic chemotherapy with carboplatin plus paclitaxel or with carboplatin alone [2, 3]. The mainstay of primary treatment is maximal cytoreductive surgery with the goal of complete resection [4]. According to a recent study among patients with stage III epithelial ovarian cancer, the addition of HIPEC to interval cytoreductive surgery resulted in longer recurrence-free survival and overall survival than surgery alone and did not result in higher rates of side effects [5]. Despite high initial response rates, ∼23 % of patients relapse during or within 6 months after end of primary chemotherapy and 60 % relapse after 6 months [6], resulting in a therapeutic challenge. The standard approach for treating recurrent ovarian cancer is chemotherapy while surgery remains an option only for some individual patients who should be carefully selected. Conventional chemotherapy has poor response with significant side-effects such as alopecia, peripheral neurotoxicity, nausea and myelosuppression. Clinical surrogate for predicting response to chemotherapy in women with recurrent ovarian cancer has been the “platinum-free interval” – that is, the period of time from cessation of primary platinum-based chemotherapy to disease recurrence [7]. As a general categorization, “platinum sensitivity” refers to disease recurrence 6 months or more after prior platinum-containing chemotherapy, and “platinum resistance” refers to a response to platinum-based chemotherapy followed by relapse less than 6 months after chemotherapy is stopped.

Platinum resistant ovarian cancer is linked to unfavourable prognosis with poor survival [8]. During that time, PM significantly compromises the quality of life, with typical and common symptoms such as ascites, abdominal pain, malnutrition, nausea, emesis, and bowel obstruction [9]. The goals of treatment should be to improve quality of life by extending the symptom-free interval, by reducing symptom intensity, and by increasing progression-free interval, and, if possible, to prolong life. Paracentesis for ascites relief, supportive care and systemic chemotherapy are treatment options offered to most patients [10]. Women with platinum-resistant disease have uniformly low response rates to chemotherapy and their benefit over best supportive care is not proven. Monotherapy is usually considered because no advantage appears to accrue to the use of non-platinum-containing combination chemotherapy.

The options for treatment include single agent chemotherapy with best supportive care, or using a range of multi-agent regimens in aggressive therapy for asymptomatic patients. Chemotherapy with, taxanes, anthracyclines, gemcitabine, topotecan, and trabectedin in various combinations and sequences are typically used. The association of non-platinum monotherapy with bevacizumab, followed by maintenance has been approved recently based on a landmark trial [11]. A Cochrane systematic review of trials in platinum-resistant EOC found that paclitaxel, pegylated liposomal doxorubicin and topotecan showed minimal benefit with different toxicity profiles [12]. No evidence supports the use of more than one line of chemotherapy in patients with platinum-resistant recurrence. Therefore, willingness of patients to undergo new therapies for modest gains is growing among these patients.

Pressurized IntraPeritoneal Aerosol Chemotherapy (PIPAC) is a novel technique delivering normothermic chemotherapy into the abdominal cavity as an aerosol under pressure. This concept seems to enhance the effectiveness of intraperitoneal chemotherapy by taking advantage of the physical properties of gas and pressure by generating an artificial pressure gradient and enhancing tissue uptake and distributing drugs homogeneously within the closed and expanded peritoneal cavity. PIPAC does not induce significant neither liver or renal toxicity [13] nor gastrointestinal symptoms [14]. Recently, two open-label, single-arm phase 2 trials performed to assess the activity of PIPAC in platinum-resistant ovarian cancer showed high local tumour concentrations of doxorubicin (4.1 μmol/g) with PIPAC with objective tumour response according to Response Evaluation Criteria in Solid Tumours (RECIST) criteria, PM Index improvement, limited hepatic and renal toxicity and tumour regression on histology [15, 16].

The rationale for the present study is because majority of patients with platinum resistant ovarian PM relapse, resulting in a therapeutic challenge. The standard approach for treating these patients is conventional chemotherapy which has poor response with significant side-effects. With these limited options, there is a need for alternative method of treatment for this group of patients. PIPAC is a safe, feasible, and tolerable palliative treatment option for selected patients with platinum-resistant ovarian cancer PM. There is an immediate need to evaluate role of PIPAC in these select group of patients as there is no similar corresponding studies.

## Materials and methods

This is a protocol of ICH-GCP phase 3, monocentric randomized trial evaluating the therapeutic efficacy measured as objective tumour response according to RECIST criteria in two groups of 50 patients each diagnosed with isolated peritoneal metastasis in women with recurrent platinum resistant ovarian cancer: an experimental group treated with PIPAC alone, a control group treated with systemic palliative chemotherapy [17].

We aimed to assess the objective tumour response of PIPAC in comparison with systemic intravenous chemotherapy for women with platinum-resistant ovarian cancer in this open-label, double arm randomized study. The study includes patients diagnosed with PM secondary to platinum-resistant ovarian cancer. All patients who were not eligible for cytoreductive surgery and HIPEC were randomized either to PIPAC group or systemic IV chemotherapy group.

### Hypothesis of study

PIPAC is a safe, feasible, and tolerable treatment for patients with platinum-resistant ovarian cancer PM, with a potentially similar or higher efficacy (objective tumour response) compared to systemic chemotherapy.

### Trial population

Consecutive patients diagnosed with PM secondary to platinum-resistant ovarian cancer who met the inclusion and exclusion criteria.

### Randomization

Stratified block randomization will be done before the initiation of the treatment (stratification based on number of lines of chemotherapy, surgical history and PCI to reduce the bias in both the groups).

### Eligibility criteria

#### Key inclusion criteria

1. Platinum resistant – have completed at least one line of chemotherapy in addition to the adjuvant regimen;
2. Age>18 years;
3. ECOG performance status 0–2;
4. No indication for CRS and HIPEC;
5. Informed consent.

#### Key exclusion criteria

1. A history of allergic reaction to platinum containing compounds or doxorubicin;
2. Ileus/obstruction;
3. Extraperitoneal systemic metastasis, including retroperitoneal disease such as aortic/para-aortic lymph nodes;
4. Renal impairment, defined as GFR<40 mL/min (Cockcroft-Gault equation);
5. Myocardial insufficiency, defined as NYHA class>2;
6. Impaired liver function defined as bilirubin ≥1.5×UNL (upper normal limit);
7. Inadequate haematological function defined as ANC ≤1.5×10^9^/L and platelets ≤100×10^9^/L.

Pertinent demographic and surgical data are prospectively recorded. Performance status was assessed according to the Eastern Cooperative Oncology Group (ECOG). Intraoperative data included peritoneal cancer index (PMI) [17], ascites (mL), adhesiolysis and operative time (min). Postoperative hospital stay and 30-day complications were recorded. The number of patients who completed all three PIPAC procedures and six cycles IV chemotherapy are noted. The quality of life assessment by Global Health Function Score and Symptom scores of EORTC QLQ-C30(Version 3.0) questionnaire will be performed before starting therapy and at 60, 120 and 180 days after first intervention. The therapeutic efficacy measured as objective tumour response according to RECIST criteria in two groups will be done by dedicated onco-radiologists. In case of CA125-only recurrences or in case the CT/MRI turns out to be wrong and you do not see peritoneal carcinomatosis upon the 1st PIPAC, they are excluded from the study and do not receive any Systemic Chemotherapy or PIPAC.

### Study assessment and time points

The response assessment by Response Evaluation Criteria in Solid Tumours (RECIST) criteria is performed at two-time points of 12 weeks and 20 weeks using MRI scanning [13].

### Primary endpoint

Proportion of patients who had an objective tumour response according to RECIST version 1.1 criteria.

### Secondary endpoints

**Figure 1:**
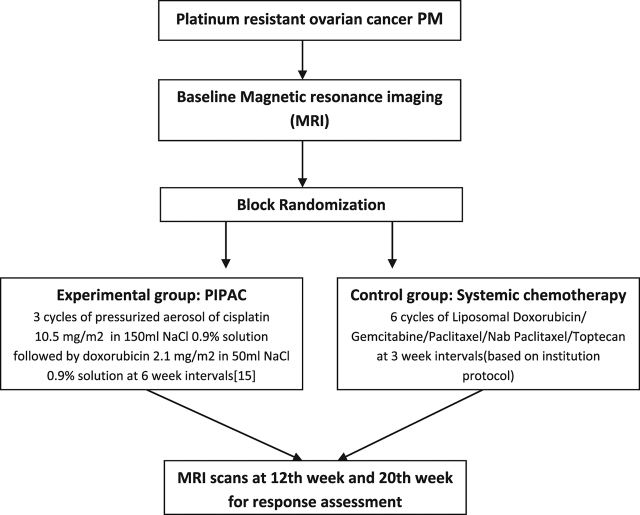
Experimental and control group including time point and technique of randomization.

(a) The number of patients who completed all three PIPAC procedures and six cycles IV chemotherapy.

(b) The proportion of patients with a deterioration of Global Health Function Score of more than 10 points 60, 120 and 180 days of EORTC QLQ-C30 after first intervention (Figure 1).

#### PIPAC group treatment algorithm

Surgical setup, treatment regimens, and safety checklist were adopted from recommendations by Solaß et al. [13, 18]. Three PIPAC treatments are scheduled at 6-week intervals after randomization. CA 125 levels and routine blood investigations are performed prior to and after each procedure. Patients are subjected to pressurized aerosol of cisplatin 10.5 mg/m^2^ in 150 mL NaCl 0.9 % solution followed by doxorubicin 2.1 mg/m^2^ in 50 mL NaCl 0.9 % solution. Aerosol flow rate of 30 mL/min and maximal upstream pressure was 200 psi with the therapeutic capnoperitoneum maintained for 30 min [16].

**Figure 2:**
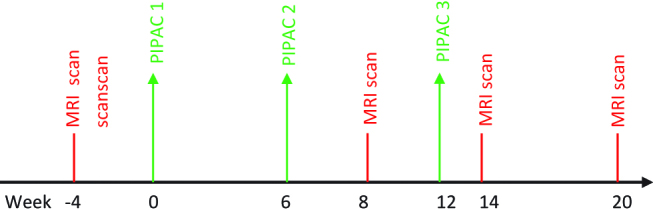
Treatment algorithm for Pressurized IntraPeritoneal Aerosol Chemotherapy (PIPAC) group.

Systematically, thoracic and abdominal magnetic resonance imaging (MRI) is performed for assessing the response. The baseline MRI scan is 4 weeks prior to first PIPAC followed by at 8th week and 14th week. A fourth MRI is scheduled at 20 weeks after finishing PIPAC therapy. Every patient is seen in outpatient consultation 4 weeks after each PIPAC procedure for monitoring of complications and evaluation (Figure 2). Reaction to treatment and side effects after each application is noted and graded as per Common Terminology Criteria for Adverse Events (CTCAE) version 4.0 [19]. The surgical complications will be recorded and grading will be done using Clavien Dindo classification system [20]. All the patients completed three cycles of PIPAC and histological response assessment was performed by an oncopathologist by the Peritoneal Regression Grading Score.

PIPAC treatment is scheduled as repeated application (3 procedures) at 6-week intervals. Thoracic and abdominal magnetic resonance imaging (MRI) is performed 4 weeks prior to therapy, in between at 8 weeks, 14 weeks and after completion at 20 weeks to assess objective tumour response with RECIST criteria.

#### Systemic IV Chemotherapy treatment algorithm

**Figure 3:**
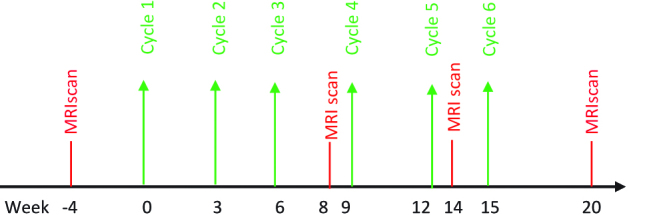
Treatment algorithm for intravenous (IV) chemotherapy group.

Chemotherapy drugs, treatment regimens, and safety checklist are decided based on the primary pathology and history of previous chemotherapy. Six systemic IV chemotherapy treatments are scheduled at 3-week intervals after randomization. Systemic chemotherapy drugs and regimen will depend on oncologist discretion and institution protocol. Systematically, thoracic and abdominal MRI is performed at 4 weeks prior to first PIPAC followed by at 8th week and 14th week. A fourth MRI is scheduled at 20 weeks after completion of treatment. Every patient is seen in outpatient consultation prior to start of each cycle with CA 125 and routine blood investigation for monitoring of complications and evaluation to proceed with further chemotherapy. (Figure 3). Reaction to treatment and side effects after each cycle is noted and graded as per CTCAE [19].

IV chemotherapy treatment is scheduled as repeated application (6 cycles) at 3-week intervals. Thoracic and abdominal magnetic resonance imaging (MRI) is performed 4 weeks prior to therapy, in between at 8 weeks, 14 weeks and after completion at 20 weeks to assess objective tumour response with RECIST criteria.

### Statistical analysis

A descriptive statistical analysis is carried out with intention to treat and quantitative and qualitative data described according to mean (± standard deviation), medians (range) and percentages. Collected data will be entered in excel software and analysed using R software version 3.4.4. Continuous variables are presented as mean with standard error of the mean or median with range or interquartile range as appropriate. Categorical variables will be presented as count and per cent. Comparison of primary endpoint RECIST criteria between two groups will be done using *χ*^2^-test. All p-values of less than 0.05 are considered statistically significant.

Objective tumour response is primary endpoints for efficacy as prediction of clinical outcomes. The Response Evaluation Criteria in Solid Tumours (RECIST) will be standard for determining the tumour response. RECIST criteria defines objective tumour response on MRI scan imaging after repeated therapies as “respond”, “stable”, or “progression”. Multivariable regression analysis will be used to detect independent variables which predict objective tumour response in the control and experimental groups [18]. Adverse events will be recorded and graded according to the Common Terminology Criteria for Adverse Events (CTCAE) version 4.0 [19]. All mortality events during treatment will be noted. Survival will be calculated in a Kaplan–Meier survival curve. SPSS 22 for Windows (SPSS Inc., Chicago, IL, USA) used for statistical analyses.

### Sample size

Sample size was calculated considering progressive disease in RECIST criteria. In PIPAC group progressive disease is expected to be 40 % and in IV Chemotherapy progressive disease is expected to be 55 %. Following formula for the sample size n was used:

$$n=(Z_{\alpha /2}+Z_{\beta }{)}^{2}\times (p_{1}(1-p_{1})+p_{2}(1-p_{2}))/(p_{1}-p_{2}{)}^{2},$$

where *Z_α_*_/2_ (level of significance=5 %) is the critical value of the normal distribution at *α*/2, *Z_β_* (power=80 %) is the critical value of the normal distribution at *β* and *p*_1_ (40 %) & *p*_2_ (55 %) are the expected sample proportions of the two groups.

Calculated sample size is 170. Considering 20 % dropout rates, sample size will be 204. For each treatment group sample size of 102 cases will be considered [21].

### Outcome measures

#### Primary outcome measures

1. Objective Tumour Response: The objective tumour response according to Response Evaluation Criteria in Solid Tumours (RECIST) criteria version 1.1.

#### Secondary outcome measures

1. Morbidity.
2. Disease-specific survival (months between inclusion and death due to ovarian cancer).
3. OS (months between inclusion and death due to any cause).
4. CA 125 levels.

### Ethical approval and consent

Institutional Review Board approval for an off label use program of PIPAC in women with PM was obtained. Institutional Review Board number: ECR/34/KA/2013/RR-16; Date of approval: 04/May/2018; Reference number: REF/2018/08/021223.

## Results and discussion

PIPAC is safe, easy to perform, and well tolerated. It is associated with histological response and increase in quality of life. Whether or not PIPAC can become a standard therapeutic option in the setting of palliative recurrent ovarian cancer treatment remains to be seen. Given the platinum-resistant nature of these patients, PIPAC protocols with other chemotherapy drugs such as taxanes, topotecan, and gemcitabine need to be investigated. Further studies using molecular targets during multiple PIPAC cycles may be useful approaches, which should be explored in clinical studies. Comparative clinical trials testing the efficacy of PIPAC when given concurrently with systemic chemotherapy are also warranted.
